# Every Man His Own Horse Doctor

**Published:** 1895-01

**Authors:** 


The fifth edition of Armatage's *Every Man His Own Horse Doctor*, with Blaine's *Veterinary Art*, has just been issued. Enlarged, partly rewritten and strengthened in many ways, it still has its place in veterinary literature. Its descriptive part and illustrations are still worthy of perusal and examination. While not destined as a text-book, it, with many others of a similar character, lessens in value with each successive edition, and in these days, when greater special technical skill is hourly demanded, the worth of such a volume, designed to make every horse owner his own horse doctor, becomes more problematical.

Works of this character will always be sought by veterinarians for consultation and to review from time to time those forms of diseases which are more or less infrequent and to familiarize one's self with the views and conclusions of Armatage, whose recognition as a skilled veterinarian has been generously conceded.

